# The Role of Perceived Susceptibility and Collectivist Values in Support for Using Social Distancing to Prevent COVID-19 in the United States

**DOI:** 10.1177/26320770211015434

**Published:** 2021-06-19

**Authors:** Xiao Wang

**Affiliations:** 1 Rochester Institute of Technology, NY, USA

**Keywords:** perceived susceptibility, collectivist values, utilitarian beliefs, value-expressive beliefs, support for social distancing, COVID-19

## Abstract

The present investigation examined the factors that were related to U.S. residents’ support for social distancing measures (i.e., stay-at-home) that can help prevent COVID-19 infections and save lives. Relying on a survey of 387 U.S.-based participants from Amazon Mechanical Turk (MTurk), conducted in May 2020, the results revealed that perceived susceptibility and collectivist cultural values predicted their support for social distancing, both directly and indirectly. The total effect sizes were moderate and strong, respectively. In addition, instrumental attitudes were a stronger predictor of the participants’ support for social distancing than experiential attitudes and perceived behavioral control. The results contribute to the understanding of how risk perceptions, collectivist values, and various attitudes are related to an important preventive behavior (i.e., social distancing) during a pandemic. It should be acknowledged that the concept of social distancing evolved throughout the pandemic in the United States.

COVID-19, a disease caused by SARS-CoV-2 virus, was initially identified in December 2019 in Wuhan, China. On January 20, 2020, the United States reported its first confirmed COVID-19 case: a man returned from Wuhan (e.g., Holshue et al., 2020). By January 31, 2021, the number of COVID-19 cases and deaths in the United States exceeded 27 million and 440,000, respectively (Coronavirus Resource Center, 2021). As SARS-CoV-2 virus spreads through respiratory droplets, keeping a six-foot distance from others and staying at home (i.e., social distancing) can help prevent infections.^1^ Since March 2020, various states in the United States have instituted some forms of stay-at-home or semi-lockdown measures (Taylor, 2020). However, media reports have shown that many people downplay the risk of contracting SARS-CoV-2 and do not follow state guidelines or mandates, which contributes to the further spread of the virus (Stenson, 2020). On the other hand, stay-at-home and semi-lockdown measures can carry an emotional and social toll (NBC News, 2020; Nicola et al., 2020) and can be difficult to practice consistently for many (van Rooij et al., 2020).

Decades of theorizing on preventive health behaviors emphasize the importance of risk perceptions and communicating risks to the public (e.g., Rosenstock, 1974). Several scholars have proposed strategies to communicate with the public about the risks associated with COVID-19 (e.g., Aven & Bouder, 2020). Research published shortly after the beginning of the COVID-19 pandemic, although informative and timely, focused on the antecedent factors that predicted risk perceptions (e.g., personal knowledge and trust in science; Dryhurst et al., 2020) or used single-item risk measures (e.g., de Bruin & Bennett, 2020). Stay-at-home or semi-lockdown measures require collective public support to be successful. Much commonsense thinking has been cast on the noncollectivist culture in the United States and how it can hinder the efforts to prevent the spread of COVID-19. Preventive behaviors, in the case of COVID-19, are only effective when people act collectively.

The relationships among these variables can be much more complicated than previously investigated. The literature on health behavior (e.g., Fishbein & Ajzen, 2010; Rosenstock, 1974) has shown that a multitude of variables (e.g., attitudes toward a health behavior and subjective norms), in addition to risk perceptions and collectivist values, determine one’s support for and practice of preventive behaviors (e.g., social distancing). Thus, are risk perceptions and collectivist values still related to preventive behaviors when other variables are considered and controlled for? Are the relationships between the two variables and public support for preventive behaviors direct or mediated by other variables? Guided by an extended version of the theory of planned behavior (TPB; Ajzen, 1991; Fishbein & Ajzen, 2010), the present research aims to examine the factors that contribute to U.S. residents’ support for the stay-at-home mandate and the associated practice of social distancing.

## The Original Version of the TPB

The TPB (Ajzen, 1991) has been widely adopted to examine the factors that predict health behaviors (Armitage & Conner, 2001; Fishbein & Ajzen, 2010; McEachan et al., 2016). The TPB states that individuals’ behaviors are predicted by intentions, which in turn are predicted by attitudes (i.e., favorable or unfavorable evaluation of a behavior), subjective norms (i.e., perceived pressure or approval of performing a behavior), and perceived behavioral control (i.e., perceived ability or confidence in performing a behavior). The main structural relationships in this theoretical framework have cumulated much empirical evidence (Armitage & Conner, 2001; Fishbein & Ajzen, 2010). In general, attitudes are the strongest predictor of behavioral intentions, and norms are the weakest (Armitage & Conner, 2001).

**Figure 1.**
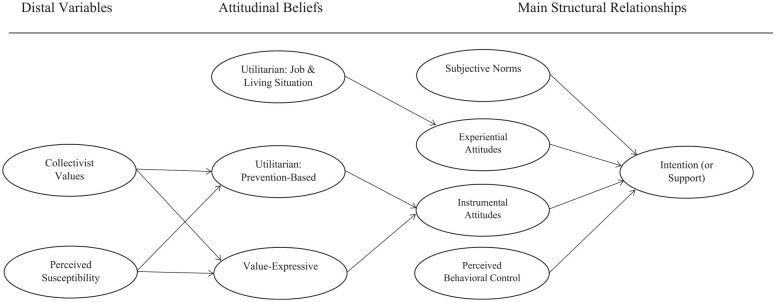
A working model of the proposed relationships among different groups of variables.

The TPB (Fishbein & Ajzen, 2010) further states that attitudes, subjective norms, and perceived behavioral control are predicted by their respective beliefs, which in turn are predicted or influenced by distal variables including risk perceptions, personality variables, socio-economic status, or media campaigns. Building on the TPB with additional theorizing on attitudes, risk perceptions, and collectivist cultural values, a working model is proposed and explained below (Figure 1).

## Experiential and Instrumental Attitudes

One fairly recent approach toward the attitudinal construct is to classify attitudes as experiential and instrumental attitudes (Fishbein & Ajzen, 2010). Experiential attitudes refer to the evaluation of the experience of performing a behavior, whereas instrumental attitudes refer to the evaluation of the outcome of performing a behavior. Fishbein and Ajzen (2010) reviewed previous research and found that semantic differential-based attitudinal items often loaded on two different factors. For example, experiential attitudes are measured by items such as enjoyable/not enjoyable and boring/not boring. Instrumental attitudes are measured by items such as good/bad and wise/not wise. Fishbein and Ajzen’s (2010) classification of experiential and instrumental attitudes is based on the content validity of the semantic differential items. McEachan et al. (2016) found that experiential attitudes were a stronger predictor of behavioral intentions than were instrumental attitudes.

However, the semantic differential items do not reveal the exact nature of these attitudes or offer practitioners good guidance regarding the specific reasons that motivate a behavior (O’Keefe, 2015; Wang, 2012, 2013). As a solution, Fishbein and Ajzen (2010) recommend the use of a belief solicitation procedure to identify the beliefs that underlie the target population’s behavioral intentions. Researchers first ask some participants, who are similar to those in the main project, to discuss or write down the beliefs that they think are related to the behavior under investigation. These beliefs are then included in the survey questionnaire. After collecting survey responses, researchers analyze which of the beliefs underlie the target audience’s behavioral intentions. Although the use of a belief solicitation procedure is important, it is not theory-based.

## Utilitarian and Value-Expressive Beliefs

Guided by attitude functional theory (Katz, 1960), Wang (2012) proposed that beliefs underlying attitudes can form different dimensions based on the functions (or goals) that they serve, including utilitarian and value-expressive functions. Attitude functional theory (Katz, 1960) stated that people are motivated to hold attitudes to serve various functions. Several functions were proposed in the literature, including a utilitarian function, a value-expressive function, and an ego-defensive function^2^ (Katz, 1960). Related to this research, the utilitarian function focuses on the basic and utilitarian aspect of an issue or a product such as the taste of ice cream or the medical efficacy of aspirin. The value-expressive function refers to how a product or an issue can help individuals express their values and identity; for example, wearing a university cap is one way to express one’s identity of being a student in a given university. Beliefs toward an object or issue can reflect the functions that the object serves. This approach is consistent with Pratkanis’ (1989) theorizing that beliefs “could be specified in multi-attribute terms by means of correlated attributes and clusters of beliefs” (p. 74). Furthermore, Wang (2013) found that the utilitarian beliefs can be further classified into two different utilitarian functions. For example, related to condom use, Wang found that the utilitarian functions of condom use can be prevention-based and enjoyment and sensation-based. This more refined classification of the utilitarian function will be considered in the present research.

## Risk Perceptions: Perceived Susceptibility

Perceived susceptibility, defined as individuals’ subjective estimate of the likelihood that a negative consequence (i.e., COVID-19) will happen to them, is considered a precursor to preventive health behavior (Janz & Becker, 1984). The health belief model states that individuals appraise risks, which together with perceived effectiveness of preventive behaviors, perceived barriers of performing the behaviors, and self-efficacy, influence their intention to engage in protective behavior (Janz & Becker, 1984; Rosenstock, 1974). Early meta-analyses showed that the relationship between perceived susceptibility and behavioral intentions was weak (*r* = .15 in Harrison et al., 1992; *r* = .19 in Floyd et al., 2000). Brewer et al. (2007) found that the bivariate relationship between perceived susceptibility to contracting a disease and vaccination behaviors was .24 based on 12 studies. Brewer et al. (2007) selected research based on whether the bivariate relationship was reported and did not consider other variables (e.g., costs or benefits of performing a behavior). Previous research has also shown that risk perceptions and behavioral intentions can be rather limited or negative. It is possible that if the proposed behavioral option is perceived to be ineffective, those who have a higher-risk perception (vs. low) will be no more likely to adopt the behavior.

However, Fishbein and Ajzen (2010) stated that because the number of beliefs and distal variables (e.g., risk perceptions) was many, they did not specify the specific relationships among these variables. Based on Wang (2013), because beliefs can be meaningfully categorized into clusters (e.g., two utilitarian belief clusters and one value-expressive belief cluster), the number of variables and relationships between distal variables and belief clusters is reduced. The relationships will be presented in the hypotheses section.

## Collectivist Values

Another variable that may predict individuals’ risk perceptions or support for social policy and behavior is their orientation toward collectivist values (i.e., collectivism; Dake, 1991; Douglas & Wildavsky, 1982). Collectivism has been conceptualized in different ways and has several dimensions (e.g., Realo et al., 1997; Triandis & Gelfand, 1998). For example, Triandis and Gelfand (1998) specified two collectivism dimensions: horizontal collectivism (among peers) and vertical collectivism (between parents/authority and the individual). On the contrary, Realo et al. (1997) proposed a hierarchical collectivism concept based on various criterion groups: family, peers, and society.

The present research focuses on the core meaning of collectivism: whether an individual gives greater priority to the group (i.e., peer group or society) than the individual (Triandis & Gelfand, 1998). In general, collectivists (i.e., those who score higher on collectivism) are more likely to be kind to and trusting of people (Shin & Park, 2005). They try to contribute to their community and group, cooperate with others, and engage in group work (Realo et al., 1997; Triandis & Gelfand, 1998). Furthermore, they favor preventive behaviors and government policies that promote collective benefits more than noncollectivists (McCarty & Shrum, 2001). Noncollectivists put themselves first and do not favor policies that restrict their personal freedom. They tend to evaluate preventive behavior or policies less effectively than collectivists (McCarty & Shrum, 2001).

## Hypotheses

Based on the literature of the TPB (e.g., Armitage & Conner, 1991; Fishbein & Ajzen, 2010), it is expected that U.S. residents’ support for social distancing measures will be a function of experiential attitudes, instrumental attitudes, subjective norms, and perceived behavioral control (H1). The present research further examines whether the more detailed belief clusters, risk perceptions, and collectivist values predict support for social distancing among U.S. residents.

In the event of SARS-CoV-2, individuals can evaluate social distancing measures, including semi-lockdowns or stay-at-home orders, based on the functions that social distancing measures serve (Katz, 1960; Wang, 2013). First, SARS-CoV-2 has infected more than 27 million people and claimed more than 440,000 lives in the United States as of January 31, 2021 (Coronavirus Resource Center, 2021). The Centers for Disease Control and Prevention (2020) recommends that social distancing can help prevent the spread of the virus and help save lives, thus serving a utilitarian function of disease prevention. Second, social distancing is restrictive and prevents people from living their normal daily lives, having a regular job, and attending school (e.g., Nicola et al., 2020). As such, these measures can carry a physical and emotional toll on the public and cause loss of employment or living situation-related issues. Third, social distancing is a “public good,” particularly for infectious diseases that may impact society as a whole, and those who do not practice social distancing may be considered reckless and irresponsible (Cato et al., 2020). Thus, practicing social distancing and following the stay-at-home orders is more than saving others’ lives; it is also one way to express one’s values of being responsible and to care about others, thus serving a value-expressive function. As the first two belief clusters (e.g., prevention and value-expressive functions) reflect instrumental attitudes and the last one (e.g., unemployment and living with social distancing) reflects the experience of going through social distancing (i.e., experiential attitudes), they should predict their respective attitudes (H2).

If the public perceives higher risks, they are more likely to endorse the utilitarian function of preventive behaviors (i.e., social distancing measures as a way to prevent the risks) and to perform such preventive behaviors as a way to express their values (H3), which are then indirectly related to instrumental attitudes. Furthermore, measures that require social distancing impose collective obligations to mitigate and prevent COVID-19 infections. Based on the discussion in the previous section, because those who score higher on collectivism value collective benefits and cooperation, they are more likely to trust that social distancing measures are effective in preventing COVID-19 than those who score low on this dimension (H4a). Collectivists also value collective measures as ways to express one’s identity of being responsible and caring (H4b). Taken together, the following hypotheses are proposed:

**Hypothesis 1 (H1):** Participants’ support for social distancing is predicted by (a) instrumental attitudes, (b) experiential attitudes, (c) subjective norms, and (d) perceived behavioral control.
**Hypothesis 2 (H2):** (a) Prevention-based utilitarian beliefs and value-expressive beliefs predict instrumental attitudes toward social distancing, whereas (b) job and living situation-based utilitarian beliefs predict experiential attitudes toward social distancing.
**Hypothesis 3 (H3):** Perceived susceptibility to risk is positively associated with (a) prevention-based utilitarian beliefs and (b) value-expressive beliefs.
**Hypothesis 4 (H4):** Collectivist values are positively associated with (a) prevention-based utilitarian beliefs and (b) value-expressive beliefs.

## Method

An online survey was conducted between May 2 and May 7, 2020. According to a timeline compiled by the *New York Times* (Taylor, 2020), 265 million Americans had been ordered or urged to stay home by March 30, 2020. On May 2, the United States reported a total of 1.1 million positive COVID-19 cases and 65,645 deaths (Coronavirus Resource Center, 2021). By early May, stay-at-home orders in a limited number of states were lifted or expired (e.g., Alabama, Alaska, Indiana, Georgia, and Montana). For the remaining states, the stay-at-home orders were in effect or replaced by less strict “safe-at-home” orders by the time this research was conducted. Most states started to enter a phased, limited reopening in May 2020 (Taylor, 2020) after this research was conducted.

### Sample

The sample was recruited from MTurk. MTurk is a crowdsourcing marketplace where individuals perform research-related tasks for companies or researchers for compensation. Tasks include, but are not limited to, responding to survey questionnaires, participating in online experiments, transcribing audio or video footages, and coding data.

Because the purpose of this research focused on U.S. residents’ support for social distancing measures, the sample parameter was set to “at least U.S. high school graduate” for participants to qualify. Furthermore, only those with IP locations in the United States were included. To exclude participants who did not answer the questions attentively, three attention-check questions were included.^3^ Among the 440 U.S. participants, 387 participants answered all three attention-check questions correctly and were retained for the final analysis. Sociodemographic characteristics of the sample are presented in Table 1. This project was approved by the Human Subjects Office at Rochester Institute of Technology. Informed consent was provided before the participants filled out the questionnaire.

**Table 1.** Sociodemographic Characteristics of the Sample.

Social demographic characteristic	Statistic
	*M* (*SD*)
Age	41.5 (13.9)
Annual income (US$)	47,131 (28,941)
Year of formal education	14.9 (4.0)
Political orientation^a^	4.6 (1.83)
	%
Gender	
Female	56.7
Male	43.1
Other/nonbinary	0.3
Race	
Asian	8.7
Black	4.4
Hispanic	5.6
White	79.0
Other racial background	2.3
Geographic area^b^	
California	11.1
Pennsylvania	7.2
Texas	6.2
Florida	6.2
New York	5.4
Ohio	5.4
Illinois	4.9
Employment status/occupation^c^	
Unemployed	13.7
Retired	7.8
Self-employed	3.1
Information technology	5.9
Sales	5.2
Accounting	2.1

*Note*. *N* = 387.

a Scale values ranged from 1 (*strong Republican*) to 7 (*strong Democrat*). ^b^Percentages of participants from other states ranged from 0% (i.e., Maine and South Dakota) to 3.1% (i.e., Georgia). ^c^Examples of other occupations included attorneys, educators, librarians, law enforcement, office assistants, and students.

### Measures

The questionnaire contained several measures and sociodemographic questions. Questions related to this analysis are listed in Table 2 and described below. Responses ranged from 1 (*strongly disagree*) to 5 (*strongly agree*). Means and standard deviations of the measures are presented in Table 3.

**Table 2.** Confirmatory Factor Analysis and Standardized Factor Loadings of Measurement Items of the Variables.

Factor and scale item	Standardized factor loading
Risk susceptibility	
Suppose there had not been any social distancing measures (e.g., stay-at-home orders; closing schools and shops)	
. . . the chance of me getting the coronavirus would be high	.92
. . . the chance of my family getting the coronavirus would be high	.92
. . . the chance of my neighbors getting the coronavirus would be high	.91
. . . the chance of people in the U.S. getting the coronavirus would be high	.78
Collectivist values	
Everyone in our country has equal responsibility	.61
Everyone should contribute their part to their group	.84
We need to cooperate with others	.86
Everyone should make some sacrifices for a better world	.71
Utilitarian beliefs—job and living situation-based	
Social distancing measures (e.g., stay-at-home orders; closing schools and shops)	
. . . can lead to (or have led to) economic issues for me (e.g., loss of income)	.79
. . . hurt my job opportunities	.70
. . . lead to living situation problems (e.g., more people/conflicts in a house)	.62
. . . lead to lower living standards	.49
Utilitarian beliefs—COVID-19 prevention	
. . . can help save others’ lives	.91
. . . can help prevent me from getting the virus	.88
. . . can help prevent my family from getting the virus	.88
. . . can help reduce the risk of getting the virus	.90
Value-expressive beliefs	
Following social distancing measures (e.g., stay-at-home orders)	
. . . shows I’m a responsible person	.88
. . . shows I care about others	.89
. . . allows me to express my values	.70
Experiential attitudes	
Regarding the social distancing measures (e.g., stay-at-home order; closing schools and shops)	
. . . they are inconvenient	.74
. . . they are boring	.74
Instrumental attitudes	
. . . they are wise	.87
. . . they are correct	.88
. . . they are beneficial	.90
. . . they are important	.92
Subjective norms	
My family expects me to practice social distancing	.90
My neighbors expect me to practice social distancing	.67
My local government expects me to practice social distancing	.55
Perceived behavioral control	
It is easy for me to practice social distancing	.69
I am confident I can practice social distancing	.76
It is difficult to practice social distancing in my neighborhood (reverse coded)	.58
My economic situation makes it difficult for me to practice social distancing (reverse coded)	.65
My job makes it difficult for me to practice social distancing (reverse coded)	.65
Support for social distancing	
I support social distancing in my state	.90
I support further social distancing to prevent more COVID-19 cases	.88
I practice social distancing	.82
I try my best to practice important health precautions in preventing coronavirus	.85

*Note*. *N* = 387. Confirmatory factor analysis showed a good fit to the data: χ^2^(584, *N* = 387) = 1,477.9, *p* < .001, root mean square error of approximation (RMSEA) = .063, 90% CI of RMSEA [.059 ~ .067], comparative fit index = .92, and standardized root mean residual = .061.

**Table 3.** Means, Standard Deviations, Pearson Correlations of the Variables Included in the Analysis.

Variable	*M*	*SD*	1	2	3	4	5	6	7	8	9	10
1. Perceived susceptibility	3.70	1.10	—									
2. Collectivist values	4.24	0.71	.29**	—								
3. Utilitarian beliefs: job/living situation-based	3.41	0.92	.08	−.03	—							
4. Utilitarian beliefs: prevention-based	4.34	0.85	.44**	.61**	−.06	—						
5. Value-expressive beliefs	3.94	0.92	.37**	.61**	−.03	.68**	—					
6. Experiential attitudes	3.61	1.05	−.03	−.14**	.38**	−.11*	−.13**	—				
7. Instrumental attitudes	4.25	0.85	.43**	.65**	−.08	.80**	.69**	−.12*	—			
8. Subjective norms	4.18	0.73	.25	.51**	−.04	.53**	.52**	−.02	.56**	—		
9. Perceived behavioral control	2.98	0.52	.10	−.03	.30**	−.10	−.03	.11*	−.07	−.03	—	
10. Support for social distancing	4.34	0.82	.46**	.69**	−.09	.79**	.67**	−.18**	.80**	.54**	−.06	—

*Note*. *N* = 387. Values for all scales ranged from 1 (*strongly disagree*) to 5 (*strongly agree*).

* *p* < .05. **p < .01.

Perceived susceptibility to contracting COVID-19 was measured by four items. These items were based on the likelihood of contracting SARS-CoV-2, similar to the wording used in the definitions by Rosenstock (1974) and Brewer et al.’s (2007) conception of the likelihood of contracting a virus: “Suppose there hadn’t been any social distancing measures (e.g., stay-at-home orders; close schools and shops), the chance of me/my family/my neighbors/people the U.S. getting the coronavirus would be high.” Individuals who scored higher on this measure perceived higher susceptibility than those who scored low on this measure. Alpha reliability was .93.

Collectivist values reflected the concept of giving greater priority to the group than to the individual and focused on what individuals should do to be contributing and cooperating members of a group or society (Realo et al., 1997). This concept was measured by four items, constructed based on similar items from Triandis and Gelfand (1998) and Realo et al. (1997): “Everyone in our country has equal responsibility,” “everyone should contribute their part to their group,” “we need to cooperate with others,” and “everyone should make some sacrifices for a better world.” Those who scored higher on this dimension value group goals more than individual goals. Alpha coefficient was .83.

Belief items are topic and situation-specific; that is, beliefs toward social distancing are different from beliefs toward a different subject matter (e.g., tweeting presidential debates). Fishbein and Ajzen (2010) advocate the selection of topic-relevant beliefs by asking participants to list their beliefs using “a free-response format” (p. 100). Five participants, who were not part of the main survey, provided a list of nine unique beliefs, which encompassed economic issues, jobs, living situation problems, COVID-19 prevention, and expressing one’s values (see Table 2). The researcher also examined the literature on the items used for measuring utilitarian and value-expressive beliefs (e.g., Shavitt, 1990; Wang, 2013). That is, belief measures were constructed using “a free-response format” and based on the definitions in the literature.

Utilitarian beliefs were individuals’ beliefs about the basic functions that social distancing would bring to them and were measured by the following items: “The social distancing measures (e.g., stay-at-home orders; closing schools and shops) can lead to (or have led to) economic issues for me (e.g., loss of income),” “. . . hurt my job opportunities,” “. . . lead to living situation problems (e.g., more people/conflicts in a house),” “. . . lead to lower living standards,” “. . . can help save others’ lives,” “. . . can help prevent me from getting the virus,” “. . . can help prevent my family from getting the virus,” and “. . . can help reduce the risk of getting the virus.” The first four items formed the first utilitarian belief cluster (i.e., job and living situation-related), and the last four items formed the second utilitarian belief cluster (i.e., prevention-based). Those who scored higher on these two dimensions held stronger concerns about employment and living situations and stronger beliefs about the preventive aspect of social distancing, respectively. Alpha coefficients were .75 and .94, respectively.

Value-expressive beliefs were defined as beliefs that performing social distancing was a way to express one’s identity and were measured by three items adapted from Wang (2013): “Following the social distancing measures (e.g., stay-at-home orders) shows I’m a responsible person,” “. . . shows I care about others,” and “. . . allows me to express my values.” Alpha coefficient was .85.

Experiential and instrumental attitudes were measured by items adapted from Fishbein and Ajzen (2010). For experiential attitudes, participants responded to two items that measured their evaluation of their experience of social distancing: “Regarding the social distancing measures (e.g., stay-at-home order; closing schools and shops), they are inconvenient/boring.” Alpha coefficient was .70. Higher scores mean less favorable experiential attitudes.

For instrumental attitudes that measured participants’ evaluation of the outcome of social distancing, participants responded to four items: “Regarding the social distancing measures (e.g., stay-at-home order; closing schools and shops), they are wise/correct/beneficial/important.” Alpha coefficient was .94. Higher scores meant more favorable instrumental attitudes.

Subjective norms were defined as the perceived pressure from people or entities that are important to the participants and measured by three items adapted from Fishbein and Ajzen (2010): “Regarding the social distancing measures (e.g., stay-at-home orders), my family/my neighbors/my local government expects me to practice social distancing.” Alpha coefficient was .75.

Perceived behavioral control was measured by five items, reflecting the participants’ ability to practice social distancing: “Regarding the social distancing measures (e.g., stay-at-home orders, closing schools or shops), it is easy for me to practice social distancing,” “. . . I am confident I can practice social distancing,” “. . . it is difficult to practice social distancing in my neighborhood,” “. . . my economic situation makes it difficult for me to practice social distancing,” and “. . . my job makes it difficult for me to practice social distancing.” The last three items were reverse coded. Higher scores meant a higher ability to practice social distancing. These items were constructed following Fishbein and Ajzen (2010) and reflected both internal and external control. Alpha coefficient was .79.

Support for social distancing refers to one’s readiness to perform a behavior (i.e., “willingness, behavioral expectation, and trying”; Fishbein & Ajzen, 2010) and was measured by four items: “I support social distancing in my state,” “I support further social distancing to prevent more COVID-19 cases,” “I practice social distancing,” and “I try my best to practice important health precautions in preventing coronavirus.” Alpha coefficient was .91.

Additional questions related to where the participants lived, an estimated number of people that they knew got COVID-19, their demographic information, and their political philosophy were included at the end of the questionnaire.

## Results

Confirmatory factor analysis and structural equation analysis were used to confirm the construct validity and to examine the relationships among the variables, respectively. For these analyses, satisfactory fit indexes are as follows (Kline, 2016): The upper bound of the 90% CI of root mean square error of approximation (RMSEA) should be less than .08, comparative fit index (CFI) should be greater than .90, and standardized root mean square residue (SRMR) should be less than .08. A large sample size can increase χ^2^ and result in a *p* value of less than .05. That is, although the *p* value for χ^2^ is preferred to be greater than .05, it is not required because the *p* value is often influenced by the sample size.

A confirmatory factor analysis was performed in the first step. Based on the maximum likelihood estimate in EQS, the model showed satisfactory fit statistics: χ^2^(*584, N* = 387) = 1,477.9, *p* < .001, RMSEA = .063, 90% CI of RMSEA [.059 ~ .067], and CFI = .92, SRMR = .061. Two utilitarian belief clusters were confirmed: One was related to job and living situations, and the other one was related to COVID-19 prevention. All items loaded on their respective factors, and standardized factor loadings ranged from .55 to .92, except for one loading of .49 (Table 2). This demonstrated the construct validity of the items used to measure each concept.

After demonstrating good construct validity, a full structural equation model analysis was performed. The full structural analysis combined both a measurement model (i.e., confirmatory factor analysis) and structural path analysis of the relationships among the variables. The model in Figure 1, with originally hypothesized relationships (H1–H4), showed a marginally satisfactory fit: χ^2^(602, *N* = 387) = 1,690.6, *p* < .001, RMSEA = .068, 90% CI of RMSEA [.064 ~ .072], and CFI = .898, SRMR = .072. Some direct paths were then added (see additional relationships below). The final structural model analysis showed acceptable fit statistics: χ^2^(602, *N* = 387) = 1,606.3, *p* < .001, RMSEA = .066, 90% CI of RMSEA [.062 ~ .070], and CFI = .91, SRMR = .066. As model Akaike information criterion (AIC) for the original model (474.6) was more than 10 points greater than that for the final model (402.3), the final model was considered a better model to capture the relationships (Burnham & Anderson, 2010). Results from the structural path analysis were used to test the hypotheses. Standardized direct, indirect, and total relationships are presented in Table 4.

**Table 4.** Standardized Direct, Indirect, and Total Effects of the Independent Variables on Support for Social Distancing.

Variable	Support for social distancing		
	Direct effect	Indirect effect	Total effect
Perceived susceptibility	.12***	.10***	.22***
Collectivist values	.34***	.38***	.73***
Utilitarian beliefs: job/living situation-based		−.03*	−.03*
Utilitarian beliefs: prevention-based	.18**	.15***	.33***
Value-expressive beliefs		.04^†^	.04^†^
Experiential attitudes	−.07*		−.07*
Instrumental attitudes	.32***		.32***
Subjective norms	−.01		−.01
Perceived behavioral control	.10*		.10*

*Note*. *N* = 387.

† *p* < .10. *p < .05. **p < .01. ***p < .001.

H1 stated that participants’ support for social distancing was predicted by instrumental attitudes, experiential attitudes, subjective norms, and perceived behavioral control. The results showed that instrumental attitudes (β = .32, *p* < .001) and perceived behavioral control (β = .10, *p* = .027) positively predicted public support and willingness, whereas experiential attitudes (β = −.07, *p* = .023) negatively predicted their support. Subjective norms were not a significant predictor (β = −.01, *p* = .850).

H2 focused on the belief clusters that predicted instrumental and experiential attitudes. Prevention-based utilitarian beliefs (β = .46, *p* < .001) and value-expressive beliefs (β = .11, *p* = .064) positively predicted instrumental attitudes, whereas job and living situation-related beliefs positively predicted experiential attitudes (β = .45, *p* < .001). Additional structural equation analysis showed that these belief variables did not cross-predict the other attitudes. Furthermore, prevention-based utilitarian beliefs were positively, directly related to support for social distancing (β = .18, *p* = .004).

H3 focused on perceived susceptibility to COVID-19. Perceived susceptibility positively predicted prevention-related utilitarian beliefs (β = .21, *p* < .001) and value-expressive beliefs (β = .17, *p* < .001). The effect sizes were rather modest.

For H4, collectivist values were positively related to prevention-related utilitarian beliefs (β = .73, *p* < .001) and value-expressive beliefs (β = .74, *p* < .001). This indicated that those with stronger collectivist values were more likely to focus on disease prevention and value expression. Collectivist values were negatively related to experiential attitudes (β = −.15, *p* = .015); that is, those with stronger collectivist values would be less likely to experience negative experiential attitudes. However, the relationship was of small effect size.

### Additional Relationships

**Figure 2.**
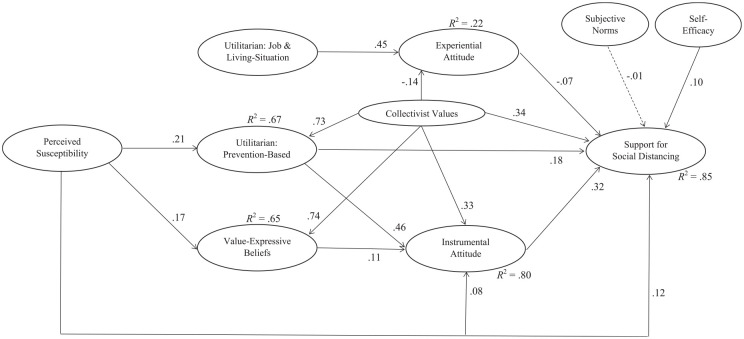
Structural equation modeling analysis of factors that predicted support for social distancing. *Note*. *N* = 387. All items loaded on their respective factors and standardized factor loadings ranged from .55 to .92 (not shown). Solid lines indicate statistical significance (*p* < .05). The full structural equation model showed acceptable fit statistics: χ^2^(602, *N* = 387) = 1,606.3, *p* < .001, root mean square error of approximation (RMSEA) = .066, 90% CI of RMSEA [.062 ~ .070], and comparative fit index = .91, and standardized root mean residual = .066.

There were several significant, direct relationships from perceived susceptibility and collectivist values to instrumental attitudes and intentions to perform social distancing. These relationships are listed in Figure 2. In general, the direct relationships between perceived susceptibility and instrumental attitudes and between perceived susceptibility and support for social distancing were in the same direction, but were weak (β = .08, *p* = .018; β = .12, *p* < .001, respectively). The direct, positive relationship between collectivist values and instrumental attitudes and support was of medium effect size (β = .33, *p* < .001; β = .34, *p* < .001, respectively).

Overall, the total effects of perceived susceptibility and collectivist values on intentions to perform social distancing were .22 (*p* < .001) and .73 (*p* < .001), respectively. That is, participants who perceived higher susceptibility to COVID-19 and had higher collectivist values were more likely to support social distancing measures. The amounts of variance explained by the direct predictors are presented in Figure 2.

## Discussion

The present investigation examined factors that contributed to U.S. residents’ support for social distancing measures. More specifically, this investigation examined beliefs that were related to instrumental and experiential attitudes and the role of perceived susceptibility and collectivist values on their intention to perform social distancing. This investigation contributes to the literature by examining the predictors of U.S. residents’ support for social distancing during a severe pandemic that requires government intervention. In contrast, previous TPB research on health behaviors (for a review, see Fishbein & Ajzen, 2010) focused mainly on individuals’ adoption of a voluntary behavior (e.g., healthy eating and physical activities).

### Theoretical Discussion

First, the present investigation contributes to the literature by providing a nuanced, yet parsimonious account of the attitudinal variables that predicted U.S. residents’ support for social distancing. The results showed that instrumental attitudes were a much stronger, positive predictor of U.S. residents’ support for social distancing, whereas experiential attitudes only weakly predicted their support. Furthermore, it shows that the prevention-related utilitarian beliefs and value-expressive beliefs positively predicted U.S. residents’ instrumental attitudes, whereas job and living situation-related utilitarian beliefs predicted experiential attitudes. In comparison, perceived behavioral control was a positive, but weak predictor of U.S. residents’ support for social distancing, whereas subjective norms did not predict their intentions.

The results were intriguing given that the media widely reported that job and living situation-related utilitarian beliefs and the ability to endure social distancing were important factors that caused the public to respond negatively toward social distancing measures (e.g., Nicola et al., 2020; Schwartz, 2020). Furthermore, most residents in the United States have a short-term time orientation (Hofstede, 1991) and enjoy “the moment.” As a result, they are less willing to sacrifice short-term benefits for long-term goals. That is, U.S. residents, in principle, favor a positive short-term experience over a positive long-term outcome of a preventive measure. The present investigation based on MTurk presents some preliminary evidence and indicates that the job and living situation-related utilitarian beliefs and experiential attitudes are less important, compared to the prevention-based utilitarian beliefs and instrumental attitudes, in predicting support for social distancing measures. Furthermore, the mean score for prevention-based utilitarian beliefs was higher than that for job and living situation-based utilitarian beliefs (*M* = 4.34, *SD* = 0.85 vs. *M* = 3.41, *SD* = 0.92). This should be further investigated with a nationally representative sample in order to understand whether the job and living situation-related utilitarian beliefs are more salient for certain economic groups (i.e., those whose jobs and living situations are more influenced by the pandemic).

Second, the present investigation provides convergent and discriminant evidence for instrumental and experiential attitudes. Following Pratkanis (1989) and Wang (2013), the present research revealed three belief clusters toward the social distancing measures. Results showed that end result-related belief clusters (e.g., prevention and value expression) positively predicted instrumental attitudes and that the experience-related belief cluster positively predicted experiential attitudes. Additional analysis showed that these belief clusters did not cross-predict experiential and instrumental attitudes. This supports the claims made by Fishbein and Ajzen (2010). Although they stated that attitudes could be classified as experiential and instrumental attitudes, their conclusion was based on factor analysis and qualitative analysis of the meaning of the semantic differential items. The construction validity of these items was not previously established. That is, this research provided both convergent and discriminant validity of experiential and instrumental attitudes, whereby they correlated highly with related concepts and did not correlate with unrelated concepts.

Third, after controlling for other variables (i.e., attitudes and perceived behavioral control), the total effects of perceived susceptibility and collectivist values on support for social distancing were .22 and .73, whereas the direct effects of these two variables on support for social distancing were .12 and .34. Of note, collectivist values appear to be a much stronger predictor than perceived susceptibility in predicting both prevention-based utilitarian beliefs and value-expressive beliefs. Considering the total effects, the effect sizes for the collectivist values were large, whereas the effect sizes for perceived susceptibility were of medium size. This indicates that for a prevention measure that requires a collective effort, collectivist values are more important than perceived susceptibility, although the role of perceived susceptibility cannot be ignored.

Finally, using belief clusters, the present research specified the relationships among these belief clusters and two distal variables (i.e., perceived susceptibility and collectivist values). According to Fishbein and Ajzen (2010), perceived susceptibility and collectivist values should be considered as distal variables and their influence is mediated by beliefs that underlie the attitudes. However, this investigation showed that perceived susceptibility and collectivist values were, directly and indirectly, related to support for social distancing. Similarly, prevention-based utilitarian beliefs should predict attitudes directly and support for social distancing indirectly. The present research observed such indirect effects, supporting the theoretical predictions. However, it also observed direct effects of these three variables on support for social distancing. There may be two possible reasons. First, the previous TPB theorizing misspelled the role of perceived susceptibility and collectivist values. In addition to being indirect predictors, they can also be directly related to the outcome variable. For example, the health belief model (Rosenstock, 1974) treats perceived susceptibility at the same level of outcome expectations and self-efficacy; that is, perceived susceptibility was conceptualized to be directly related to preventive behaviors and does not need to be mediated by attitudes toward preventive behaviors. Similarly, prevention-based utilitarian beliefs do not need to be mediated by instrumental attitudes. Second, it is possible that the measurements of beliefs and attitudes did not encompass their respective concepts and thus, they did not fully mediate the effects of distal variables. Either possibility cannot be ruled out based on the present results. Future research should consider analyzing both direct and indirect relationships.

### Practical Implications

As mentioned at the beginning of the discussion, the results were obtained through an MTurk sample. If these results are confirmed by a larger, more representative sample, it is then important to focus on the variables that strongly predict support for social distancing, that is, perceived susceptibility, collectivist values, prevention-based utilitarian beliefs, and instrumental attitudes. For example, a change of one standard deviation in U.S. residents’ prevention-based utilitarian beliefs can lead to a change of .33 standard deviation in their support for social distancing. However, strategies to address the above variables are different. For perceptions or belief-related variables, which are open to change, it is important to provide the target audience risk information and prevention knowledge to enhance their perceived susceptibility and prevention-related utilitarian beliefs. On the other hand, because collectivist values are probably formed over the years and are not easily changed, it might be important to match message appeals to participants’ value orientations (i.e., collectivist values). To be more specific, because collectivists care about collective risks and benefits, messages should focus on these aspects to encourage the collectivists to support social distancing policies. In this sample, approximately 18% of the participants had a collectivistic value lower than 3.5 on a 5-point scale. For these individuals, a strategy can be to roll back and focus on perceived susceptibility and prevention-based utilitarian beliefs. Finally, contrary to media reports that unemployment and living situation issues prevent many from supporting social distancing, the present investigation did not find this to be a major factor. It is possible that the media focused on the groups that were most affected by the pandemic, which were not well represented in this survey. This has to be confirmed by a larger, more representative sample; an additional, more targeted analysis on some social and economic groups should be conducted.

### Limitations and Future Research Directions

Several issues should be acknowledged. First, this research was conducted using MTurk. Although many scholars stated that research using MTurk can generate high-quality social science research data (e.g., Buhrmester et al., 2018), caution should be exercised when interpreting survey-based research. That is, the sample in this research was not representative of the general public in the United States. Relatedly, Table 1 shows that although some demographic characteristics of the sample were comparable to those of the U.S. population, African Americans and Latinos were underrepresented in the sample. Future research should be conducted with more representative samples to confirm or disconfirm the results. Second, data quality using an online participant pool may suffer from inattention. The present research used three attention-check questions and screened out those who failed even one of the three questions to ensure data quality. That is, there was some confidence that the participants in the final sample answered the questions attentively.

Third, it should be acknowledged that the practice and definition of social distancing changed over time. This investigation was conducted in May 2020 when social distancing meant stay-at-home orders for the majority of the states in the United States. After May or June 2020, social distancing generally means keeping a six-foot distance from others. Furthermore, facemask wearing and handwashing are also important behaviors to prevent COVID-19 infections and should be dealt with in future research. Finally, further research should include a measure of severity and investigate how perceived severity might influence Americans’ support for health measures. Additional research should also be fine-tuned.

### Conclusion

Regardless of the limitations, the present research has provided some evidence on what can be addressed and included in a public health campaign to prevent COVID-19 infections. Such insights can help elicit public support for a health measure that can help contain a highly contagious and somewhat deadly virus. Public support for and practice of recommended actions are often based on the effectiveness of the prevention measures against and perceived susceptibility to COVID-19. These are often complicated by many other variables, including their cultural values, political environments, misinformation, and their financial and living situations. At the theoretical level, the present research has tested a model that explained the direct and indirect relationships among distal variables (e.g., collectivist values and, perceived susceptibility), the intermediate variables (e.g., beliefs and attitudes), and support for social distancing, and provided some evidence that can help fine-tune the risk and attitude model.

## Notes

1 Facemask wearing and handwashing have also been recommended by the Centers for Disease Control and Prevention (2020) as means to prevent COVID-19 infections.

2 The ego-defensive function refers to how holding an attitude can help maintain one’s self-esteem or help defend one’s ego from external threats (Shavitt, 1990). Not all behaviors/issues serve all three functions. This function was not mentioned during the belief solicitation survey discussed in the method section.

3 An example of the three attention-check questions is as follows: For this question, please select “disagree.” Participants who did not pay attention to the questions were likely to miss some of the three questions. Participants who answer each question correct received 1 (wrong = 0). The average was 2.80.
